# Infertility and inequality: a plea for action

**DOI:** 10.1093/humrep/deag157

**Published:** 2026-10-06

**Authors:** Letizia Li Piani, Edgardo Somigliana

**Affiliations:** Infertility Unit, Fondazione IRCCS Ca’ Granda Ospedale Maggiore Policlinico, Milan, Italy; Department of Clinical Sciences and Community Health, Dipartimento di Eccellenza, University of Milan, Milan, Italy; Infertility Unit, Fondazione IRCCS Ca’ Granda Ospedale Maggiore Policlinico, Milan, Italy; Department of Clinical Sciences and Community Health, Dipartimento di Eccellenza, University of Milan, Milan, Italy

**Keywords:** infertility, inequality, ART, access, IVF

## Abstract

The recent global guidelines from the World Health Organization on diagnosis, prevention, and treatment of infertility invites us to consider infertility beyond the traditional framing as a clinical condition, recognizing it as a profound issue of equity. The guideline explicitly states that fertility care should be founded on equity, evidence-based information, and respect for human rights while rejecting stigma or discrimination. In this contribution, we propose a conceptual framework that interprets infertility as a system of layered inequalities operating across global, structural, social, temporal, narrative, and individual dimensions. At the global and system levels, geographical differences, legislative fragmentation, disparities in access, and public–private services imbalances define reproductive opportunities. Social and cultural factors, including unequal access to education, limited fertility awareness, biased representations, and potential stigmatization, further contribute to the marginalization of affected individuals, disproportionately impacting vulnerable and minority groups. Time represents a crucial yet often overlooked determinant of injustice. Its manifestations differ across settings, sparing from delayed access to infertility treatment in resource-limited countries to the growing mismatch between biological reproductive constraints and societal trends toward delayed parenthood in many high-income settings. In this context, technological solutions such as assisted reproduction and elective egg freezing may inadvertently reinforce disparities by shifting responsability to individuals rather than addressing broader barriers, partly sustained by misleading narratives in social media. These dynamics often weigh more on women and ultimately interact to shape the individual experience of infertility. Infertility care requires coordinated action beyond a clinical one. Embracing a public health perspective and ameliorating access to fertility services must become the priority, sustained by investment in fertility education, inclusive narratives, and family-friendly policies, alongside greater harmonization of legislation. Considering infertility as an issue of equity means addressing upstream determinants that influence reproductive opportunities long before individuals seek care. The key question is not only who can access fertility treatment, but who reaches it with a meaningful chance of success. Only through such efforts can the opportunity to pursue one’s reproductive goals and build a family, if wished, becomes a reality for many, rather than a privilege for a few.

## Introduction

In December 2025, the World Health Organization (WHO) published its first global guidelines on infertility, a milestone marking a paradigm shift in reproductive health priorities, from the prevention of unintended pregnancies to the recognition of unmet reproductive intentions. Yet beyond the clinical recommendations, one message stands out in the opening of the document: infertility is not only a medical condition, but a profound issue of equity (WHO, 2025), one of the most contemporary expressions of inequality.

It does not operate as a single phenomenon, but as a system of layered disadvantages, unfolding across global, structural, and socio-cultural dimensions. These inequalities can be understood as a system of concentric layers, each interacting with and reinforcing the others. Moving from outer structural determinants to more intimate and individual dimensions, these layers progressively shape the individual experience of infertility (Fig. 1).

**Figure 1.**
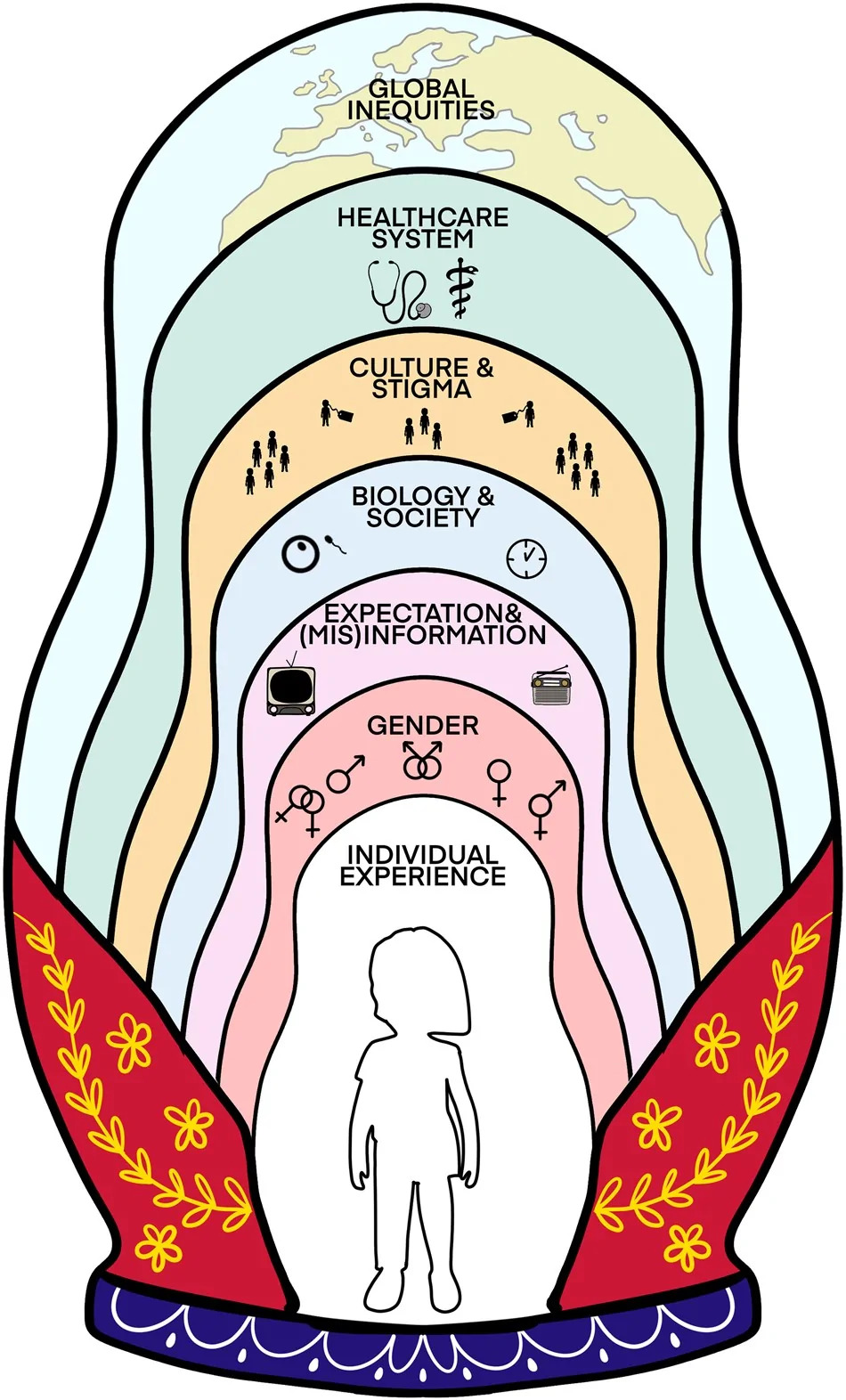
**A conceptual framework of infertility as a multi-layered system of inequities.** Conceptual framework illustrates infertility as a system of layered and interacting inequalities. From the outer to the inner layers: global inequities, healthcare system factors, socio-cultural dimensions, biological constraints, and societal dynamics, expectations, and (mis)information surrounding reproductive technologies. Time is a cross-cutting dimension. Gender is conceptualized as a multidimensional and non-binary construct that intersects across all layers. All the dimensions ultimately converge to shape the individual experience of infertility. The figure was created with Procreate app (Dr Giorgia Di Stefano).

## The outer layer: global inequities

Access to fertility care remains deeply unbalanced worldwide (Inhorn and Patrizio, 2015; Fauser *et al.*, 2024). Although the prevalence of infertility is estimated to be relatively homogeneous worldwide (WHO, 2025), this apparent epidemiological homogeneity contrasts sharply with the profound inequalities in access to diagnosis, treatment, and reproductive opportunities observed across settings. In fact, in many low- and middle-income countries, access to ART remains limited (DeWeerdt, 2020; Macklon and Fauser, 2020; Chiware *et al.*, 2021). In these settings, specific forms of infertility, such as secondary infertility or uterine infertility due to fibroids, are particularly prevalent (Ombelet *et al.*, 2025). As in these societies, fertility is still considered a marker of social value, infertility brings higher risk for stigma, marginalization, and gender violence (Stellar *et al.*, 2016; Bornstein *et al.*, 2020; Van Rooij *et al.*, 2020; Gerrits *et al.*, 2023). Although infertility inequity is universal, its expression is context-dependent (Fauser and Macklon, 2025). Even if ART has revolutionized infertility care, it has historically evolved as a highly specialized system, making access to treatment strongly based on availability of financial, organizational, and technological resources, with the greater impact on resource-limited countries (Fauser *et al.*, 2024; Norman and Fauser, 2024). On the other hand, in high-income countries, disparities are less often driven by the absence of fertility care than by unequal access to available services, shaped by legislation, funding, and healthcare organization (Fauser *et al.*, 2024). Examples include differences in eligibility for single women and same-sex couples, reimbursement policies, upper age limits for treatment, and access to donor gametes, which vary substantially across countries (Fertility Europe, 2024).

## The system layer: structural barriers to care

While global inequities characterize the geography of disparities, structural elements constitute their architecture, explaining how fertility injustice is generated and perpetuated (Macklon and Fauser, 2020; Afferri *et al.*, 2022; Fauser and Macklon, 2025; Liang *et al.*, 2025). This architecture is founded on multiple interacting factors, such as *system organization*, *economic models*, *physical accessibility*, *technological complexity*, and *legislative frameworks* (Ombelet, 2014; Fauser *et al.*, 2024; Norman and Fauser, 2024).

### System organization and economic models

No healthcare system is inherently equitable (Mladovsky and Sorenson, 2010). Insurance-based systems, such as those adopted in the USA, as well as predominantly private structures that characterize many limited-resource countries, often generate financial barriers to global fertility care access (Chambers *et al.*, 2009; Chiware *et al.*, 2021). On the other hand, public universal healthcare organizations are not necessarily immune to inequities (Messaoud *et al.*, 2023). Accessibility of fertility services is shaped by public-private balance, public investment dimensions, and reimbursement policies (Fauser *et al.*, 2019; Fauser *et al.*, 2024). As a result, insufficient public capacity may generate prolonged waiting lists, shifting access toward the private sector and inevitably privileging those who can afford to pay for ART treatment. Italy offers a paradigmatic example. Although the universal healthcare coverage, access to fertility care varies significantly across regions, with marked differences in quality, availability, and waiting times between the north and the south (Palomba *et al.*, 2024). Waiting times may extend to more than a year in some regions, forcing many patients to seek care in other regions (internal migration), in private settings or to postpone treatment (Palomba *et al.*, 2024).

In a field where time is a key prognostic factor, such delays are not merely organizational inefficiencies, but clinically relevant determinants of reproductive chances.

### Geographical accessibility

Logistical barriers, including travel distance, transportation, accommodation, and the geographical distribution of fertility centers, represent an additional structural determinant. Individuals living in rural or remote areas, such as in countries with low population density, might face a significantly higher risk of non-access to IVF/ICSI center due to long distances required to reach the centers (Harris *et al.*, 2016).

### Technological complexity

The technological evolution of ART has dramatically improved reproductive outcomes. However, it has also increased the complexity of fertility care, requiring intensive monitoring and sophisticated laboratories, expensive ovarian stimulation protocols, and highly specialized personnel (Ombelet, 2014; Fauser *et al.*, 2024). These factors not only translate into higher costs, but also encourage the concentration of expertise within a limited number of specialized centers, creating additional barriers. The recent success of a proof-of-concept of a mobile laboratory, with low-cost embryo culture and mild stimulation, might suggest that ‘complex’ model of IVF care might not be necessary for all infertile patients or every context (Ombelet and Goossens, 2016; Boshoff *et al.*, 2026). These approaches illustrate how reducing selected technological and organizational barriers could potentially broaden access, particularly in resource-limited settings.

### Legislative framework

Legislative fragmentation across countries—including differences in eligibility for single women, same-sex couples, donor gametes, reimbursement policies, and age limits—determines who can access fertility care, under which conditions, and at what cost (Fertility Europe, 2024). Therefore, laws not merely regulate access to ART, but actively define reproductive opportunities across populations. This heterogeneity has fueled the rise of cross-border reproductive care but also contributes to inadequately regulated pathways (Salama *et al.*, 2018; Whittaker *et al.*, 2019; Ethics Committee of the American Society for Reproductive Medicine, 2022). These patterns remain difficult to capture and may further exacerbate the isolation and discrimination of already vulnerable groups.

## The social layer: culture, stigma, and representation

Beyond access, infertility is deeply rooted in social and cultural disparities. A persistent lack of accurate information and fertility education contributes to widespread misconceptions about reproductive potential (Pedro *et al.*, 2018; Bettinger *et al.*, 2025; Ekstrand Ragnar *et al.*, 2025; John *et al.*, 2025).

In Italy, the 2018 National Fertility Survey highlighted a substantial gap in fertility awareness across generations (Istituto Superiore di Sanità Progetto “Studio Nazionale Fertilità”Studio Nazionale Fertilità, 2020). Although community-based services such as ‘family counseling centers’ are formally recognized as key promoters of sexual and reproductive health under national law (Law No. 40/2004; Law No. 405/1975), they remain underused and poorly known, particularly among younger populations. The absence of updated data further reflects limited political engagement on this issue. Similar community-based reproductive health services exist in many countries, yet fertility awareness is rarely among their primary priorities (Harper *et al.*, 2017, 2021; Martins *et al.*, 2024).

A narrow and often inaccurate narrative, in which stigma and silence prevail, further undermines fertility awareness. This is particularly evident in the context of gamete donation, where reluctance to disclose experiences remains common (Duff and Goedeke, 2024; Goedeke *et al.*, 2026). Many individuals and couples experience infertility in isolation, reinforcing a cycle of invisibility and minority stress (Greil *et al.*, 2010; Gameiro *et al.*, 2015). This burden is disproportionately higher among groups already exposed to discrimination, including lesbian, gay, bisexual, transgender, queer or questioning, intersex, and asexual (LGBTQIA+) individuals, single parents, and racial minorities (Goossen *et al.*, 2019; Campo-Engelstein and Quinn, 2021; Kirubarajan *et al.*, 2021; Ekstrand Ragnar *et al.*, 2025). Stigma and misconceptions may also contribute to delayed help-seeking, postponing infertility assessment, ultimately reducing the likelihood of successful treatment (Greil *et al.*, 2010; Domar *et al.*, 2021).

Digital representations also play a role in shaping perceptions (de Boer, 2021; de Boer *et al.*, 2022). As an example, while common narratives often associate Black women with higher fertility, evidence suggests they face a significantly increased risk of infertility (Jackson-Bey *et al.*, 2021).

Such misconceptions, reinforced through digital content and platforms, may contribute to delayed recognition of infertility and help-seeking among Black women. This example illustrates how social narratives and digital tools—including artificial intelligence—may perpetuate biases, if not critically designed and implemented. However, not all digital platforms pose the same risk. The quality and reliability of fertility-related information vary considerably across digital platforms, depending on scientific control, quality assurance, and potential hidden conflicts of interest (Grace *et al.*, 2023; Dhanoya *et al.*, 2025). Such variability may further amplify existing informational inequities. At the same time, digital communication might offer an innovative opportunity to *democratize* access to reproductive health information, particularly in settings where either fertility education or healthcare services remain limited. The challenge therefore does not lie in reducing digital information, but in ensuring evidence-based, high-quality, and transparent information (WHO, 2021, Global strategy on digital health 2020–2025).

## The time layer: the mismatch between biology and society

Reproductive timing represents one of the most underestimated axes of inequality, with different forms according to the area considered. While access to fertility care is often framed as the central issue, the most decisive factor frequently precedes it: access ‘in time’.

In resource-limited settings, delayed access to fertility care often results from social stigma, limited fertility awareness, geographical barriers, financial constraints, and the scarcity of specialized services (Ombelet, 2014; Inhorn and Patrizio, 2015). These factors may substantially postpone infertility assessment and treatment, ultimately reducing reproductive chances.

In contrast, in many high-income societies, time-related inequity increasingly arises from a societal trend of delayed parenthood to later stages of life, driven by education, career aspiration, economic instability, and unawareness of the fertility constraints (Sobotka, 2017; Fauser *et al.*, 2024). However, biological constraints have not evolved at the same pace of societal transformations, as highlighted by evolutionary theories (Roseman and Auerbach, 2025). This mismatch might create a structural tension, in which individuals are socially encouraged to delay reproduction, while remaining biologically constrained in doing so. Although the reasons for delaying parenthood differ across socioeconomic groups, delayed parenthood has become a widespread societal phenomenon in many high-income societies (Fauser *et al.*, 2024). Consequently, while the biological consequences of reproductive ageing affect everyone, access to technologies presented as solutions to this mismatch remains unequally distributed. Elective egg freezing (social freezing) has been presented as a solution to this pressure and depict as a tool for reproductive autonomy (Petropanagos *et al.*, 2015; Fritz and Jindal, 2018; Pennings, 2021; Teo *et al.*, 2022). Yet, its accessibility remains largely limited to those with sufficient financial resources, deepening existing disparities (Ben-Rafael, 2018). Its growing normalization (Johnston *et al.*, 2022) also raises an important ethical question: does it genuinely expand reproductive choice, or does it encourage individuals to adapt to broader social and structural constraints? In other words, is it a genuine form of empowerment, or a subtle one of *disempowerment*?

By shifting the focus from structural barriers to individual solutions, the narrative around elective egg freezing risks diverting attention from the broader determinants of delayed parenthood (Petropanagos *et al.*, 2015), while placing increasing responsibility on individuals to compensate for them. In this sense, what is often framed as empowerment could also be indeed a subtle action of unequal redistribution of responsibility from society to the individual, despite the well-established health risks associated with later pregnancies (Fritz and Jindal, 2018).

## The narrative layer: expectations and the illusion of ART success

In settings where ART is commonly available, another form of inequity emerges: not the absence of technology, but the way in which it is socially represented. The portrayal of IVF in social media and digital platforms contributes to unrealistic expectations. Although often presented as a reliable and accessible solution, IVF success rates remain limited: WHO estimates that only approximately one-third of couples undergoing IVF ultimately achieve a live birth (WHO, 2025). However, the perception of IVF success rate is not always coherent with real numbers (Devroe *et al.*, 2022; McMahon *et al.*, 2024). This discrepancy between perception and reality is not neutral: it influences reproductive decision-making, potentially favoring delayed parenthood under the assumption that technology will compensate for time. A similar misconception has already been described for elective egg freezing (Hirsch *et al.*, 2024).

This optimistic and unrealistic view might partly be grounded in the undeniable technological progress, which has impressively expanded reproductive options (Fauser *et al.*, 2019). However, it might also contribute, intentionally or not, to sustaining false expectations, not always aligned with clinical outcomes (Devroe *et al.*, 2022; McMahon *et al.*, 2024). As a result, ART risks being perceived not as one option among many, but as ‘the safety net’. When this perception replaces a realistic understanding of reproductive boundaries, it may inadvertently reinforce the very inequalities it is thought to mitigate. Unreliable information may influence individuals’ choices and enhance disparities among subjects.

## The inner layer: gendered experiences of infertility

Gender inequity remains crucial to infertility care (Fitzgerald *et al.*, 2024). Although infertility is biologically shared between partners, it continues to be predominantly perceived and managed as a female condition (Chu *et al.*, 2019; WHO, 2025). In clinical practice, women are often the primary, and sometimes the only, interlocutors in fertility pathways, while male involvement remains limited. Economical analysis aimed at evaluating the cost-beneficial ratio of infertility treatments exclude males (ESHRE Capri Workshop Group, 2015). At the same time, important gaps persist in gender-research (Duffy *et al.*, 2021; Bijloo *et al.*, 2026). Several conditions affecting female fertility, such as endometriosis, have historically been under-recognized and under-investigated (Watson, 2024). Moreover, long-term health implications of female infertility remain less explored compared with male one, further reflecting gender disparities in research priorities (Hanson *et al.*, 2017, 2018; Choy and Eisenberg, 2018; Chen *et al.*, 2022; Maiolino *et al.*, 2023; Laukhtina *et al.*, 2021; Polymeropoulou *et al.*, 2026).

Gender inequalities also intersect with broader societal structures (Ferreirinha *et al.*, 2025; Su *et al.*, 2026). The lack of strong and effective supportive family policies contributes to the burden of reproductive decision-making, particularly for women and contributes to age-related infertility (Ferreirinha *et al.*, 2025; Su *et al.*, 2026). Even in academic settings, spaces that should represent the most advanced and enlightened sectors of society, motherhood continues to be an obstacle to career progression (Costello *et al.*, 2025; Cairo *et al.*, 2026).

In this sense, gender inequity is not merely a background condition, but an upstream determinant conditioning how infertility is addressed, managed, and ultimately experienced.

## Conclusions: call to action

In this context, infertility emerges not only as a clinical condition, but as a lens through which broader societal inequities become visible (WHO, 2025). Addressing it requires coordinated action at multiple levels.

Improving access to fertility care must become a public health priority, with greater investment in equitable and accessible services. A more rational and comprehensive economic evaluation of the benefits of infertility interventions is warranted to justify additional resources rather than dogmatically begging for equity. The integration of fertility education into school curricula, abandoning the narrow focus on the prevention of unintended pregnancies, and the support of family counseling services are key steps in the promotion of ‘a fertility culture’. Infertility must be openly discussed, challenging heteronormative and traditional narratives and reducing stigma through inclusive communication. Policies must support individuals and couples in reconciling reproductive and professional aspirations. Greater harmonization of legislation and international coordination could help mitigate existing disparities. Finally, research efforts should prioritize inclusive and affordable approaches, particularly in resource-limited settings.

At its core, reproductive health concerns reproductive autonomy: the ability to pursue one’s reproductive goals, whether these involve creating a family or not. Yet the opportunities to pursue these goals are not equally distributed.

As long as fertility remains shaped by unequal access to time, resources, and information, infertility will continue to reflect, and *reproduce*, broader global and social inequities. Expanding access to care is necessary, but it is not sufficient. Without addressing the conditions that delay, discourage, or disadvantage individuals long before they seek treatment, inequality will persist upstream, where it is less visible, but more decisive. Reframing infertility as a matter of equity means shifting the focus from intervention to prevention—not only in the biological sense, but first and foremost in the social one. The question is not only who can access fertility care, but who arrives there with a meaningful chance of success. Only through these efforts can we ensure that pursuing family formation does not remain a privilege for a minority, but a real possibility for all who wish for it.
